# CD137 in tuberculosis: a scoping review of an emerging immune checkpoint at the crossroads of diagnosis, prognosis, and therapy

**DOI:** 10.3389/fimmu.2025.1682269

**Published:** 2025-11-03

**Authors:** Nuraddin Nasir Goronyo, Bih H. Chendi, Shalena Naidoo, Novel N. Chegou

**Affiliations:** South African Medical Research Council Center for Tuberculosis Research, Division of Immunology, Department of Biomedical Sciences, Faculty of Medicine and Health Sciences, Stellenbosch University, Cape Town, South Africa

**Keywords:** CD137, 4-1BB, tuberculosis, diagnostic biomarkers, prognostic biomarkers, immune activation, cytokines, immunotherapy

## Abstract

**Introduction:**

CD137 (4-1BB), a member of the tumour necrosis factor (TNF) receptor superfamily, plays a key role in T-cell activation, survival, and cytokine production, functions that are central to immune responses against *Mycobacterium tuberculosis*. This scoping review brings together current evidence on the clinical relevance of CD137 in tuberculosis (TB), including its potential as a diagnostic, prognostic, and therapeutic target.

**Methods:**

This review was conducted in accordance with the PRISMA extension for Scoping Reviews (PRISMA-ScR). Relevant studies on CD137 in TB were identified through database searches and screened using predefined eligibility criteria. Experimental, animal, and human studies reporting on CD137 expression, function, or clinical associations were included. Key information from each study was charted to describe the scope, characteristics, and main findings of the available evidence.

**Results:**

We identified ten eligible studies involving *in vitro* experiments, animal models, and human cohorts. CD137-positive T cells and soluble CD137 (sCD137) levels were consistently elevated in active TB, with some evidence suggesting the ability to distinguish disease states and predict severity. Mechanistic studies show that CD137 modulates cytokine responses, including interferon-gamma (IFN-γ) and TNF-α, and interacts with other immune checkpoints such as programmed cell death protein-1 (PD-1) and cytotoxic T-lymphocyte-associated protein-4 (CTLA-4). Preclinical models have demonstrated that CD137-targeted strategies may enhance mycobacterial control. Although current findings are promising, most studies are small, geographically limited, and exploratory.

**Discussion:**

CD137 remains an underexplored immune checkpoint with potential to inform host-directed TB diagnostics and therapies, offering a new angle for precision immunology in high-burden settings. Large-scale, longitudinal studies are needed to define its role in host immunity and determine its translational value.

## Highlights

- CD137, a co-stimulatory receptor, shows diagnostic potential for TB disease
- Elevated soluble CD137 levels in TB predict TB severity and shorter survival time.
- CD137^+^ γδ T cells significantly reduce mycobacterial growth.
- CD137-targeted immunotherapy may enhance immune response in TB treatment.
- Limited evidence: large-scale studies are needed to evaluate CD137’s clinical role.

## Introduction

Tuberculosis (TB) remains one of the top infectious disease killers globally, with an estimated 10.8 million new cases and 1.3 million deaths reported in 2023 (1). The burden is particularly high in low- and middle-income countries (LMICs), where limited diagnostic infrastructure and rising rates of drug-resistant TB further complicate disease control efforts (2). While early detection and treatment initiation are crucial for TB control, current diagnostic methods remain inadequate, particularly for extrapulmonary TB (EPTB) (3), paediatric TB (4) and TB in HIV-coinfected individuals (5).

Existing TB diagnostic tools have notable limitations (6, 7). Smear microscopy lacks sensitivity (8), GeneXpert remains expensive and inaccessible in many healthcare settings (9), and IGRAs cannot distinguish active TB (ATB) from latent infection (LTBI) (2, 10–12). While culture remains the reference standard, it is time-consuming, requires a biosafety level 3 (BSL3) environment and skilled personnel, and is not widely available in high-burden settings. As such, there is a need for alternative, more accurate and rapid diagnostic tools. Significant efforts are being made in the search for additional host-derived biomarkers that may be useful in diagnosing TB, particularly in high-burden settings. IGRAs solely depend on the detection of *in vitro* secretion of IFN-γ (which is amongst the most investigated targets) by lymphocytes after *Mycobacterium tuberculosis* (*M. tb)* antigen stimulation, providing an accurate diagnosis of TB infection (13). Hence, the increased demand for new and advanced assays capable of investigating the predictive value of alternative biomarkers that can indicate the risk of the development of ATB in individuals exposed to TB (11).

The immune response to *M.tb* involves complex interactions between innate and adaptive immunity (14). Macrophages and dendritic cells (DCs) play a key role in bacterial containment (15), while CD4^+^ and CD8^+^ T-cells contribute to IFN-γ and TNF-α secretion, which is essential for granuloma formation and bacterial clearance (16). However, the precise regulation of these responses remains poorly understood (17).

CD137 (4-1BB), an immune checkpoint receptor belonging to the tumour necrosis factor receptor superfamily (TNFRSF9) (18), has emerged as an important T-cell co-stimulatory molecule, driving immune cell activation and cytokine production in TB (19). CD137 is primarily expressed on activated CD4^+^ and CD8^+^ T cells, but can also be found on B cells, natural killer (NK) cells, dendritic cells (DCs), neutrophils, endothelial cells (20), and human microganglia (21). Upon binding to its ligand CD137L (4-1BBL, TNFSF9), CD137-CD137L interaction enhances immune activation and triggers downstream signalling pathways that upregulate cytokine production, promote T-cell memory formation, and protect T-cells from activation-induced apoptosis (22–29). As such, CD137 signalling may influence the balance between bacterial clearance and immune pathology (30). Initial studies showed that CD137 protein was strongly induced by *M.tb* in human microganglia, unlike a weaker, transient response to *M. avium*, suggesting non-transcriptional regulation (31). CD137L is critical for dendritic cell-mediated priming of CD4^+^ T cells in *M.tb* infection. Its absence resulted in multiple immunological abnormalities, including reduced IL-12 and TNF-α, increased IL-10, and poorly organised granulomas. These dysfunctions were associated with only a mild and transient impairment in bacterial control (20), suggesting that while CD137L strongly shapes the immune response, its direct impact on long-term bacterial burden remains unclear. Furthermore, soluble CD137 (sCD137) released into circulation upon T-cell activation has been associated with immune activation (32) and might be a potentially useful biomarker for TB infection. Elevated sCD137 levels also correlated with TB disease severity and treatment response (19, 33). These findings highlight CD137’s potential as a key player in TB immune and host-pathogen dynamics (34, 35). However, its precise role remains unclear, and the breadth and quality of evidence is limited, with few studies conducted in high-burden TB settings.

This scoping review maps the current evidence on CD137 in TB, with a focus on its emerging role in clinical diagnosis, prognosis, and immunotherapy. We highlight key findings, identify knowledge gaps, and propose directions for future research to advance the translational potential of CD137 in TB care.

## Methods

### Study design and search strategy

This scoping review followed the Preferred Reporting Items for Systematic Reviews and Meta-Analyses Extension for Scoping Reviews (PRISMA-ScR) guidelines. A comprehensive literature search was conducted across four electronic databases, i.e., PubMed, ScienceDirect, SCOPUS, and ClinicalTrials.gov, to identify peer-reviewed articles, systematic reviews, meta-analyses, and clinical trials investigating the role of CD137 (also known as 4-1BB or TNFRSF9) in tuberculosis (TB).

The search strategy used combinations of the following terms: (“CD137” OR “4-1BB” OR “TNFRSF9”) AND (“Tuberculosis” OR “TB” OR “Mycobacterium tuberculosis” OR “M. tuberculosis” OR “Mtb”). No date restrictions were applied, and only English-language articles with full-text availability were included. The initial search yielded 36 records from PubMed, 328 from ScienceDirect, 15 from SCOPUS, and 0 from ClinicalTrials.gov. Duplicates (n = 10) were removed using EndNote software.

### Study selection

**Figure 1:**
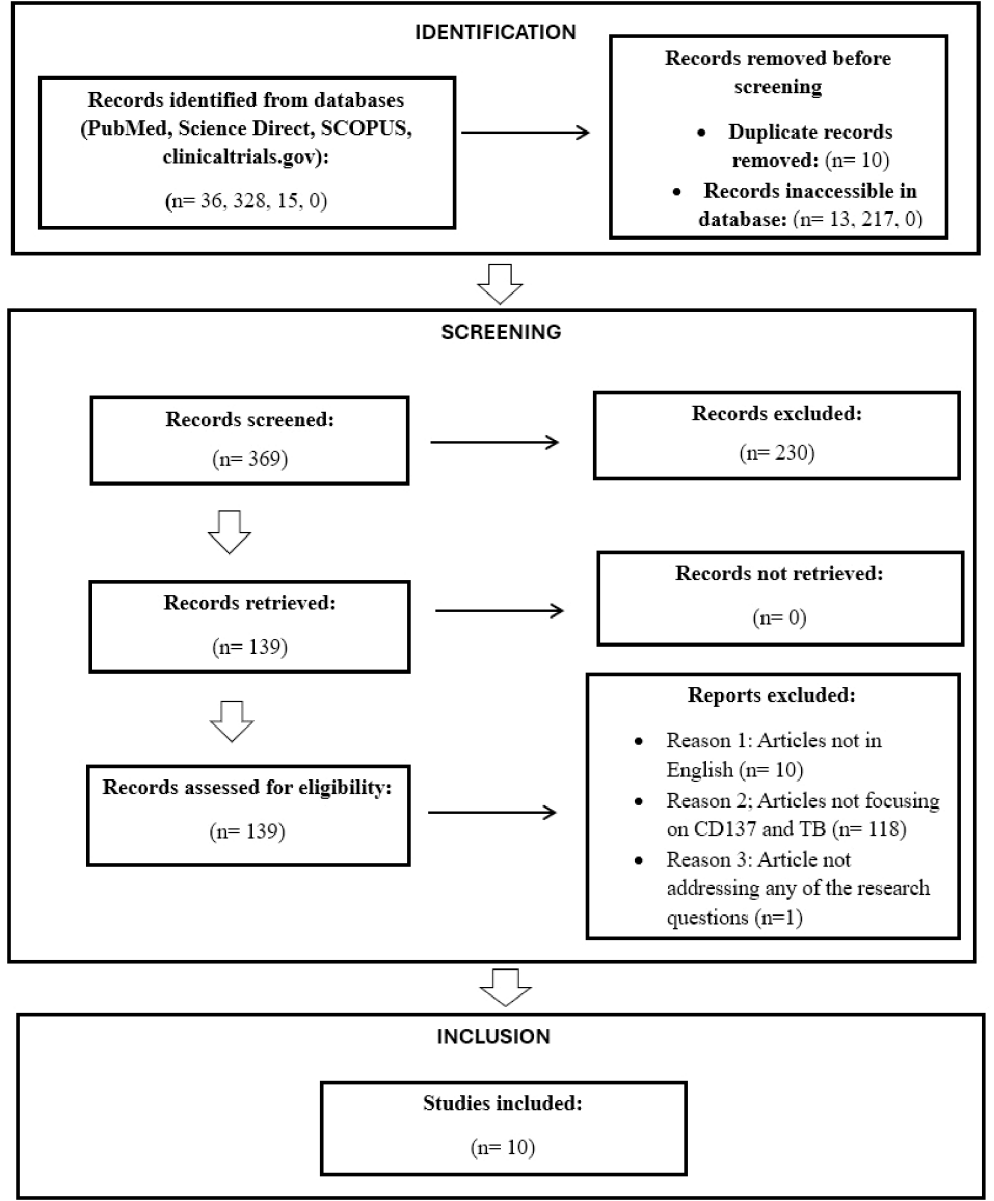
PRISMA 2020 flow diagram for selection of articles for the proposed scoping review.

Titles and abstracts were screened manually for relevance using pre-specified inclusion and exclusion criteria. Full texts of potentially eligible studies were reviewed, resulting in 10 studies that met the final inclusion criteria (as illustrated in **Figure 1**).

#### Inclusion criteria

The following were the criteria used in including articles:

- Studies published in English
- Full-text availability through the specified databases
- Articles investigating CD137 in the context of TB (human or animal models)
- Peer-reviewed original research articles (including observational and experimental studies)

#### Exclusion criteria

- Non-peer-reviewed articles (e.g. editorials, letters, or commentaries)
- Studies unrelated to both CD137 and TB
- Retracted publications or articles without accessible full text

### Data extraction and categorisation

From each included study, the following data were extracted: publication details (author, year, location), study design, population/sample, methods used to assess CD137, and key findings. Based on relevance to the review objectives, studies were categorised into five thematic domains:

1. Molecular mechanisms involving CD137 in TB pathogenesis
2. Interactions of CD137 with other immune markers in TB
3. Diagnostic role of CD137 in TB
4. Prognostic role of CD137 in TB
5. Therapeutic potential of CD137 in TB

To facilitate analysis and comparison, the results from included studies were synthesised thematically and summarised in **Tables 1**–**5**. These tables provide a structured overview of each study’s objectives, methods, findings, and relevance within their respective thematic domains. This approach allowed for a comprehensive mapping of the current evidence landscape and highlighted key gaps in knowledge to inform future research directions.

**Table 1:** Summary of studies describing the molecular mechanisms associated with CD137 in tuberculosis.

Author	Location	Study population	CD137 assessment method	Key findings
Ngar woon Kam et al., 2024 (34)	Queen Mary Hospital, Hong Kong, China	NPC patients with TB reactivation (n=2), healthy donors	Tissue mapping, PBMC stimulation with *M.tb* CFP-10:ESAT-6, assessed CD137^+^ cell density	CD137^+^ CD8^+^ T cell density in granulomas (mean: 78.5 ± 25.1); higher CD137^+^ IL-12^+^ M1 and CD137^+^ CCL17^+^ M2 densities in tuberculomas.
Carla Palma et al., 2010 (41)	Rome, Italy	C57BL/6 mice (DNA-primed NP-mice, p-mice)	Splenocyte stimulation with Ag85B, assessed IFN-γ and TNF-α with CD137 pathway blockade	CD137 blockade increased IFN-γ and TNF-α at 16h post-M. tb stimulation; reduced with PMA; increased TNF-α, decreased IFN-γ at 2–5 days.
Dario A. Fernández Do Porto et al., 2012 (30)	Hospital Muniz, Bueno Aires, Argentina	Healthy adults (n=40, BCG-vaccinated, QFT-negative, HIV-uninfected), TB patients (n=40, <1-week anti-TB therapy).	PBMC stimulation with M. tb H37Rv, assessed CD137/CD137L expression on monocytes and NK cells.	CD137 expression was induced on CD14^+^ monocytes and NK cells in both groups post-overnight stimulation.
Jin Jing et al., 2018 (38)	309th Hospital, Beijing, China	TB patients and healthy controls	PBMC and TPE stimulation with *M.tb* H37Rv lysates, measured 4-1BB expression on MAIT cells	4-1BB expression elevated on MAIT cells from PBMCs (p=0.0008) and TPE (p<0.0001); higher in TPE vs. PBMCs (p<0.0001); IL-2Rα higher on 4-1BB^+^ vs. 4-1BB^-^ MAIT cells (p=0.0078).
Julia Maria Martinez et al., 2014 (20)	National University of Singapore, Singapore	CD137L-deficient and wild-type mice.	BMDC infection with M.tb CDC1551, measured TNF-α and IL-6 levels, and CD4^+^/CD8^+^ T cells in MLNS.	Higher TNF-α (p<0.01) and IL-6 (p<0.05) in CD137L-deficient mice; fewer activated CD4^+^ T cells in MLNs at 3 weeks, higher at 10–14 weeks in CD137L-deficient vs. WT.
Monica Curto et al., 2004 (31)	School of Medicine, Cagliari, Italy	Microganglia cultures infected with M. avium MAC type 1 (SmD and SmT)	Immunostaining of CD137 in cultures infected with SmD and SmT	SmD induced CD137-positive immunostaining at 4h, decreased at 16h; SmT induced staining at 2h, decreased at 48h; no mRNA change, immunostaining intensity correlated with pathogenicity.

**Table 2:** Summary of studies describing the interactions between CD137 and other immune markers in tuberculosis.

Author	Location	Study population	CD137 assessment method	Key findings
Ngar woon Kam et al., 2024 (34)	Queen Mary Hospital, Hong Kong, China	TB patients post-pembrolizumab, healthy donors	Correlation analysis of PD-1, CD137, IFN-γ, IL-12, CCL17	Negative correlation PD-1 with IFN-γ (-0.59) and IL-12 (-0.45); positive correlation CD137 with IFN-γ (0.86), IL-12 (0.93), CCL17 (0.99).
Carla Palma et al., 2010 (41)	Rome, Italy	C57BL/6 mice (DNA-primed NP-mice, p-mice)	Agonistic anti-4-1BB mAb treatment, measured IFN-γ, IL-10, MIP-1ß, CD233	Increased IFN-γ (p<0.01), CD233 (NP-mice: 22.4 ± 1.3, p<0.01; p-mice: 15.5 +/- 0.5); decreased IL-10 and MIP-1ß (p<0.01).
Dario A. Fernández Do Porto et al., 2013 (40)	Hospital Muniz, Bueno Aires, Argentina	TB patients, healthy donors	Bayesian modelling of CD137: CD137L interactions	Indirect Bayesian model reproduces some of the data, but was outperformed by the direct signalling model (Bayes factor of 43.7 dB).
Ji et al, 2024 (39)	Shanghai Pulmonary Hospital, China	TB patients, TB resisters, and healthy controls	Assessed PD-1 and CTLA4 expression on CD137^+^ Vγ2Vδ2 T cells	Higher PD-1 (>30%) and CTLA4 (>10%) on CD137^+^ Vγ2Vδ2 T cells in TB vs. healthy controls (p<0.01).

**Table 3:** Summary of studies describing the diagnostic role of CD137 in tuberculosis.

Author	Location	Study population	CD137 assessment method	Key findings
Z.-H. Yan et al., 2017 (37)	Beijing Chest Hospital, China	TB patients, LTBI patients, and healthy controls	Flow cytometry (CD137 on CD4^+^ T cells, pre/post-CFP-10 stimulation)	CD137^+^ CD4^+^ T cells are higher in TB vs. healthy (p<0.0001), LTBI vs. healthy (p=0.0012), and TB vs. LTBI (p=0.0045). Post-stimulation increase in TB (p=0.0018) and LTBI (p<0.0001).
Yi L. et al., 2024 (19)	Beijing Chest Hospital, China	PTB patients (SE: 60, non-SE: 79), healthy controls	ELISA (sCD137 levels in blood)	sCD137 higher in TB vs. healthy (p=0.012; AUC: 0.6177, sensitivity: 42.45%, specificity: 74.68%); higher in SE vs. non-SE (p=0.0056; AUC: 0.7715, sensitivity: 60.00%, specificity: 82.28%).

**Table 4:** Summary of studies describing the prognostic role of CD137 in tuberculosis.

Author	Location	Study population	CD137 assessment method	Key findings
Yi L. et al., 2024 (19)	Beijing Chest Hospital, China	PTB patients (n=139; SE: 49, non-SE: 78, deceased: 12), healthy controls (n=80)	ELISA (sCD137 levels in blood)	sCD137 levels increased from healthy to non-SE, SE, and deceased groups (p=0.0128); high sCD137 (>43.72 pg/mL) was associated with shorter survival time (p=0.0041).

**Table 5:** Summary of studies describing the therapeutic role of CD137 in tuberculosis.

Author	Location	Study population	CD137 assessment method	Key findings
Ji et al, 2024 (39)	Shanghai Pulmonary Hospital, China	TB patients, TB resisters, and healthy controls	PBMC infection with M. tb H37Rv, adoptive transfer of CD137^+^ Vγ2Vδ2 T cells to SCID mice	Adoptive transfer reduced mycobacterial growth in macrophages (p<0.01).
Carla Palma et al., 2010 (41)	Rome, Italy	C57BL/6 mice (DNA-primed NP-mice, p-mice)	Splenocyte stimulation with Ag85B, treated with agonistic anti-4-1BB mAb	Agonistic anti-4-1BB increased IFN-γ secretion upon CD3ε engagement in NP-mice and p-mice (p<0.01).

## Results

### Overview of studies

Our initial database search across PubMed, ScienceDirect, and SCOPUS yielded 369 records, and 10 were included in the final analysis (**Figure 1**). After removing duplicates and inaccessible articles, 139 were advanced for full-text screening. An additional 129 records were further excluded for the following reasons: 118 were not focused on CD137 and TB, 10 were not written in English, and one did not align with any of our predefined research questions.

**Figure 2:**
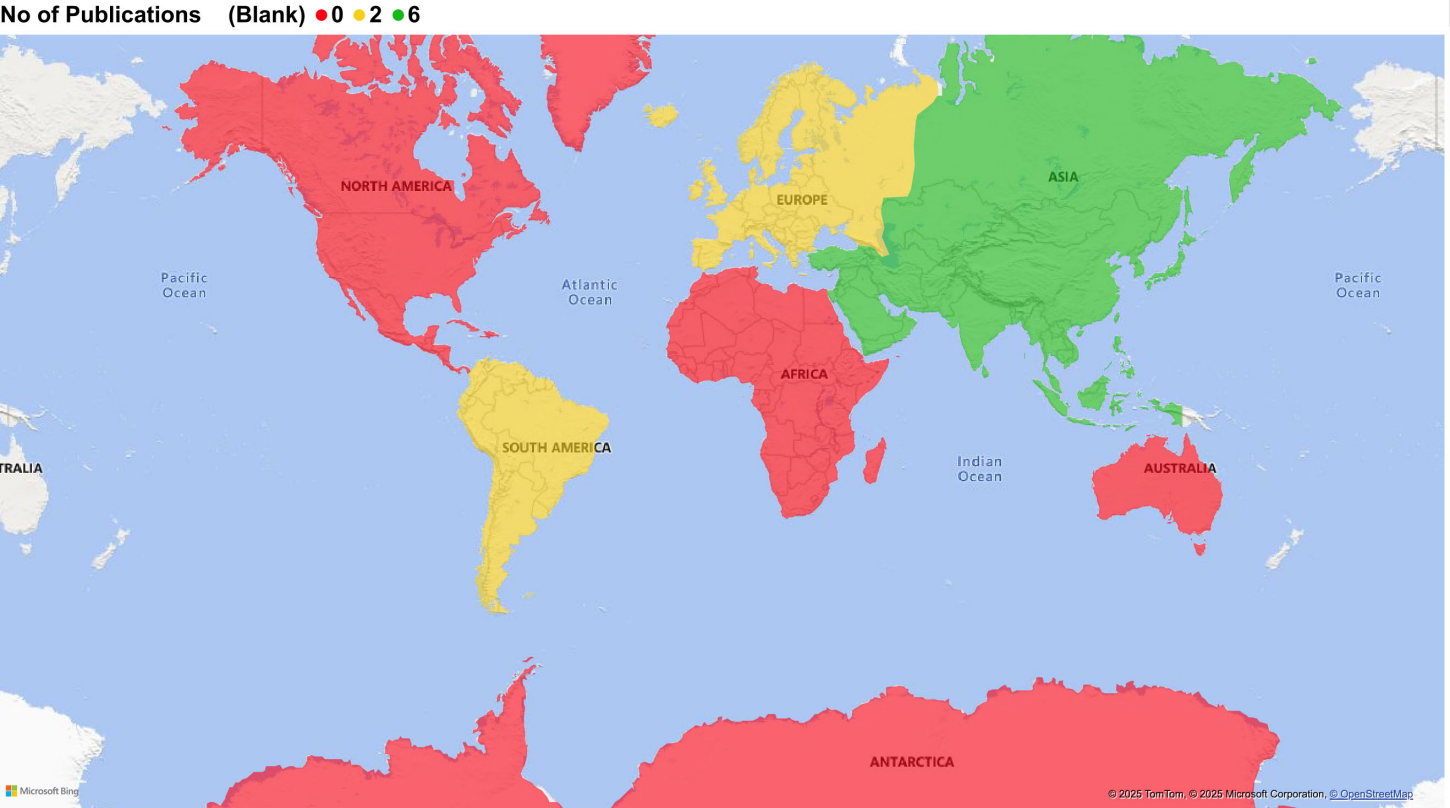
Geographical representation of CD137-research published studies. Five studies were conducted in China (Asia), two each from Argentina (South America) and Italy (Europe), and one from Singapore (Asia).

Of the 10 studies, half (n = 5) were conducted in China, and the remainder were from Argentina (n = 2), Italy (n = 2), and Singapore (n = 1), as depicted in **Figure 2**. Therefore, most research on CD137 in TB has emerged from Asia, followed by Europe and South America, with no contributions from Africa, North America, or Australia. Notably, the selected studies originated from China (Asia region), which is a country ranked among the top 30 high-TB burden countries, similar to African regions accounting for 24% of the global TB burden, with no CD137-related TB research. This geographic disparity of CD137 in TB represents a critical research gap.

We observed that three of the studies were published only recently (2024), while others were published within the past two decades, including 2004, 2010, 2012, 2013, 2014, 2017, and 2018. Although CD137 has been investigated in the context of other diseases, such as cancer (36), it only recently gained attention as a possible biomarker worth evaluating in the context of TB.

**Figure 3:**
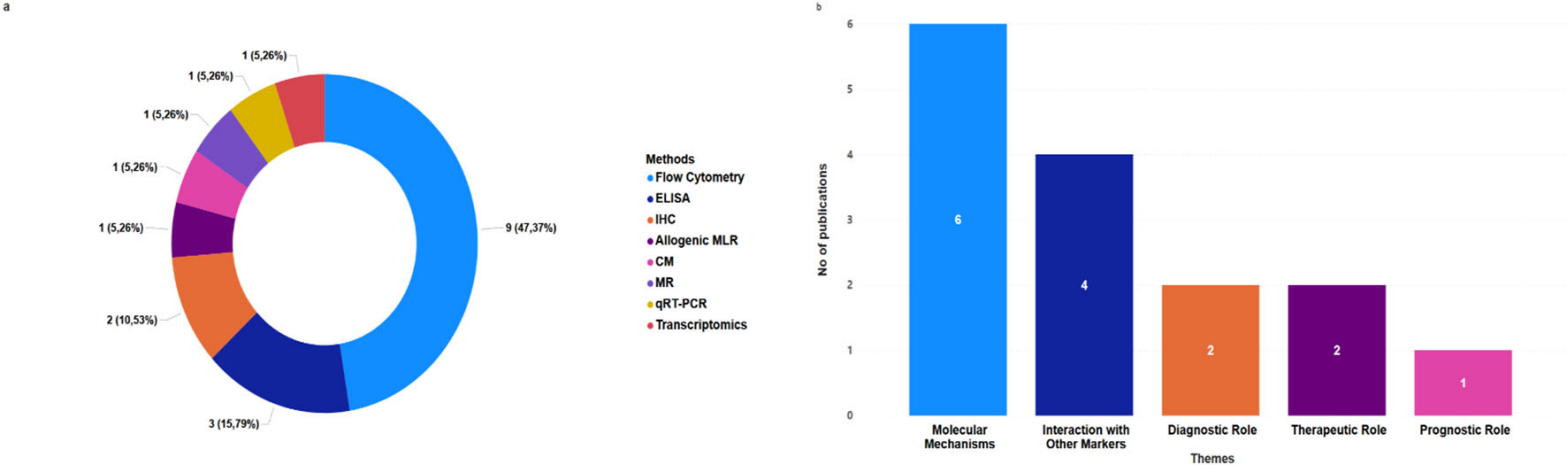
Distribution of CD137 assessment methods and research themes in TB. **(a)** Distribution of selected papers by CD137 assessment methods. **(b)** Frequency of selected publications related to each theme in TB studies.

Regarding the methodology employed in the included studies, flow cytometry and ELISA emerged as the most frequently used analytical techniques for CD137 assessment, followed by immunohistochemistry, amongst others, as summarised in **Figure 3a**. The studies employed diverse experimental approaches, ranging from *in vitro* T-cell stimulation assays (n=5) (30, 37–40) to *in vivo* murine models (n=2) (20, 41), and using clinical cohort samples (n=7) (19, 30, 37–40, 42). The sample types analysed included serum/plasma, peripheral blood mononuclear cells (PBMCs) (34, 37–40), paraffin-embedded lung tissues (34), recombinant immune cells (34), and mouse lungs (20). These diverse experimental approaches underscore the need to delineate CD137’s immunological relevance with flow cytometry and ELISA, highlighting the ability to measure both the membrane-bound and soluble forms of CD137. In alignment with our research objectives, we categorised our findings into five themes as depicted in **Figure 3b** and discussed in detail in the sections below.

### Molecular mechanisms associated with CD137 in TB

Studies investigating the molecular interactions of CD137 and TB were the most frequent among all the included studies in this scoping review (**Table 1**). Using mouse models, human PBMCs, and co-culture samples, researchers investigated how CD137 signalling modulates host immunity to *M.tb*. Key mechanisms identified include:

*CD137 and/or CD137L Expression:* A study by Fernández Do Porto et al (30) stimulated PBMCs with *M. tb* H37Rv whole-cell lysate antigens, prepared by probe sonication, from 40 healthy adults (BCG-vaccinated, QFT TB Gold-negative, HIV-uninfected) and 40 active TB patients, all of whom received less than one week of anti-TB therapy; this overnight stimulation induced CD137 expression on CD14^+^ monocytes and NK cells in both groups. Also, Monica Curto et al (31) studied human microglia cultures infected with *Mycobacterium avium* complex (MAC) type 1 strains (SmD and SmT) and with *M. tuberculosis*. Infection with SmD induced CD137-positive immunostaining after 4 hours, which decreased after 16 hours, whereas SmT induced staining after 2 hours that lasted longer but decreased after 48 hours. Strikingly, *M. tb* infection induced CD137 staining much earlier, with stronger intensity that was maintained for longer. Despite no significant change in CD137-specific mRNA levels, immunostaining intensity was enhanced, suggesting that CD137 expression correlates with pathogenicity.

*Immunopathogenesis:* Martínez Gómez et al (20) infected bone marrow-derived dendritic cells (BMDCs) from CD137L-deficient and wild-type (WT) mice with *M. tb* CDC1551. Compared to WT BMDCs, CD137L-deficient BMDCs secreted higher levels of TNF-α (p<0.01), IL-6 (p<0.05), and IL-10 (p<0.001). Functionally, BCG-infected CD137L-deficient BMDCs induced weaker proliferation of naïve T cells in mixed lymphocyte reactions. *In vivo*, *M. tb*-infected CD137L-deficient mice showed delayed activation of CD4^+^ T cells in mediastinal lymph nodes (MLNs) at 3 weeks post-infection, followed by significantly higher frequencies of activated CD4^+^ T cells at weeks 10 and 14 compared to WT mice. In contrast, no major differences were observed in total or activated CD8^+^ T cell populations. Notably, this study did not report CFU counts or lung histopathology, limiting direct conclusions on bacterial control or tissue pathology. Another study that broadly addressed this mechanism was done by Jing Jiang et al (38) and found elevated 4-1BB (CD137) expression on mucosal-associated invariant T (MAIT) cells from PBMCs (p=0.0008) and in tuberculous pleural effusions (TPE; p<0.0001) after *M.tb* H37Rv stimulation. MAIT cells from TPE showed higher 4-1BB expression than those from peripheral blood (p<0.0001). They also found higher expression of IL-2Rα on 4-1BB^+^ MAIT cells than on 4-1BB^-^ MAIT cells (p=0.0078).

*Cytokine Secretion:* Fernández Do Porto et al (30) reported that blocking the CD137 pathway increased IFN-γ and TNF-α production 16 hours after stimulation with *M. tb* H37Rv antigens in TB patients and healthy donors, whereas blocking the pathway following PMA stimulation reduced these cytokines. At 2- and 5-days post-*M.tb* antigen stimulation, CD137 blockade increased TNF-α but decreased IFN-γ. Another study by Carla Palma et al (41) studied C57BL/6 mice immunised with Ag85B-encoding DNA and boosted with Ag85B protein (NP-mice) or not boosted (P-mice). Under polyclonal CD3ϵ stimulation, anti-4-1BB enhanced IFN-γ production by both CD4^+^ and CD8^+^ T cells, consistent with its costimulatory role. Furthermore, Jing Jiang et al (38) also investigated the relationship between 4-1BB expression and MAIT cells function and found: 4-1BB^+^ MAIT cells from TPE produced more IFN-γ than 4-1BB^-^ cells (p<0.0001), with 4-1BB expression correlating with IFN-γ production (p=0.0051). Secondly, in their effort to assess the role played by IL-2 and IL-12 in 4-1BB expression, they blocked these cytokines and found that blocking IL-2 reduced 4-1BB expression (p < 0.01), whereas IL-12 blockade had no effect (p > 0.01).

*Granuloma formation:* An observational study by Kam et al. (34) studied two nasopharyngeal carcinoma patients with TB reactivation post-pembrolizumab therapy, and revealed that CD137^+^ CD8^+^ T cells were abundant in granulomatous tissues (mean density: 78.5 ± 25.1). Tuberculomas showed higher densities of CD137^+^ IL-12^+^ M1 and CD137^+^ CCL17^+^ M2 cells compared to other tissues.

**Figure 4:**
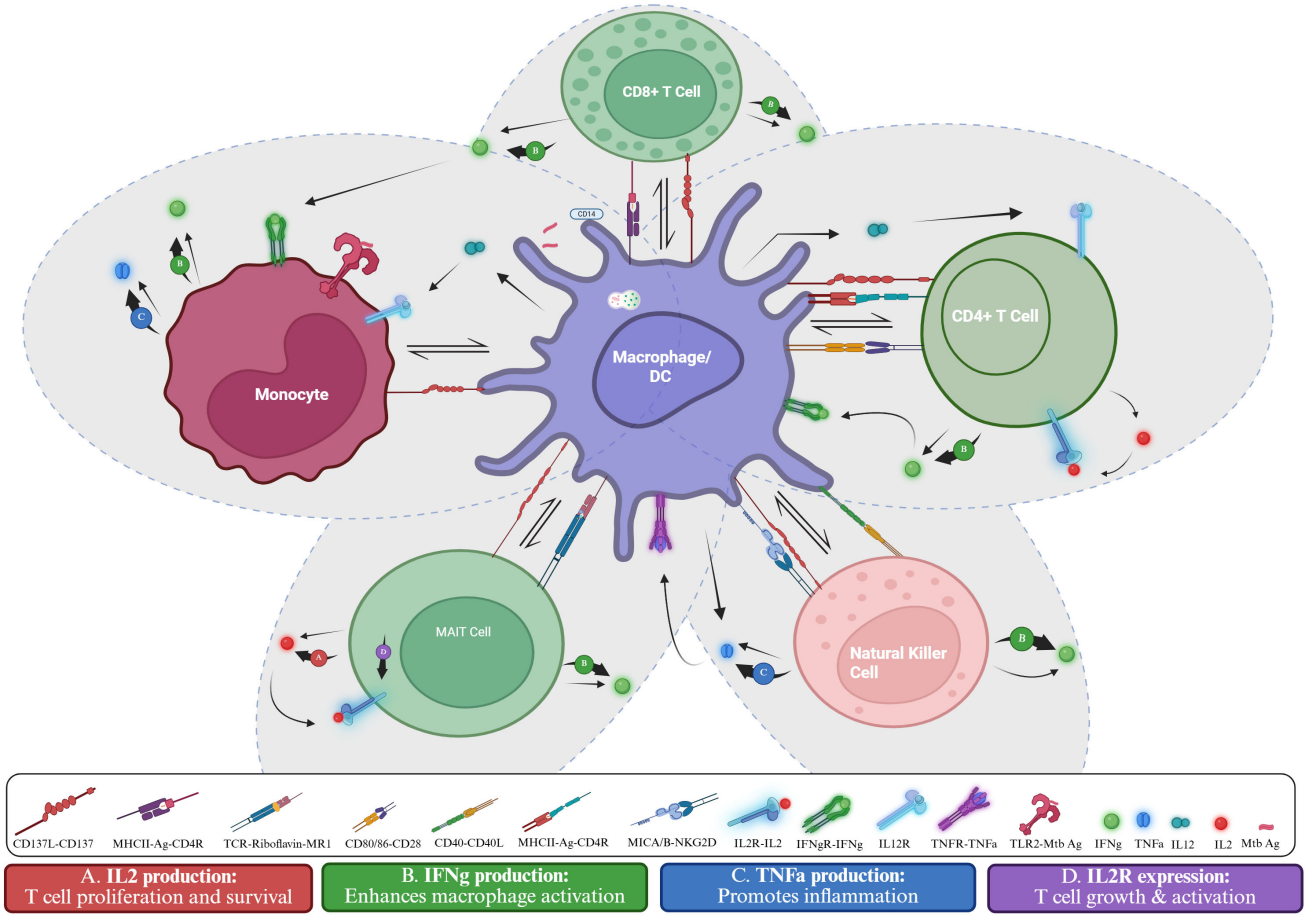
CD137-mediated immune cell and macrophage activation in Mtb infection. The schematic shows interactions among macrophages/dendritic cells, monocytes, CD4**^+^** and CD8**^+^** T cells, MAIT cells, and NK cells. Antigen presentation, and co-stimulation with receptor-ligand signals drive cytokine responses. CD137-Dependent effects: **(A)** CD137 signalling in MAIT cells promote IL-2 secretion (38), supporting T-cell proliferation and survival. **(B)** CD137 signalling in CD4^+^ (41), CD8^+^ (41), NK cell (31), MAIT cells (38), and monocytes (31), enhances IFN-γ secretion, boosting macrophage activation. **(C)** CD137 signalling in NK cells and monocytes (31) boosts TNF-α production (31) which mediates inflammation. **(D)** CD137 signalling in MAIT cells upregulates IL-2R expression (38), supporting T cell growth and activation. Created in BioRender. Nasir Goronyo, N. (2025) https://BioRender.com/guvghez.

These mechanisms highlight the function of CD137 and CD137L as both promoters and regulators of TB immunity, as illustrated in **Figure 4**. Following *M.tb* or *Mycobacterium avium* stimulation, CD137 expression patterns were linked to infection dynamics and pathogenicity. CD137L deficiency altered cytokine responses and impaired CD4^+^ T cell activation and granuloma formation, weakening bacterial control. Blocking or stimulating the CD137 pathway modulates IFN-γ and TNF-α production. CD137^+^ immune cells are also enriched in granulomatous TB lesions, suggesting a role in local immune responses.

### Interactions of CD137 with other immune markers

Several studies (**Table 2**) evaluated CD137’s interplay with other immune regulators in TB. These include:

*Checkpoint Molecule Co-Expression:* A correlation analysis performed by Ngar woon kam and colleagues (34) reported a negative association between PD-1 and IFN-γ/IL-12 (correlation index: -0.59 and -0.45) and a positive association between CD137 and IFN-γ/IL-12/CCL17 (correlation index: 0.86, 0.93, and 0.99). Meanwhile, another study by Ji et al (39) found higher PD-1 (>30%) and CTLA4 (>10%) expression on CD137^+^ Vγ2Vδ2 T cells in TB patients compared to healthy controls (p<0.01).

*Cytokine Environment Modulation*: Palma et al (41) studied C57BL/6 mice immunized with Ag85B-encoding DNA and boosted with Ag85B protein (NP-mice) or not boosted (P-mice). Upon Ag85B stimulation, agonistic anti-4-1BB treatment reduced IFN-γ secretion by CD4^+^ T cells in NP-mice (p<0.01), while no effect was observed in P-mice; this inhibition required CD8^+^ T cells and was associated with reduced expression of CD25, CD38, CD233, and CD44 on Ag85B-specific CD4^+^ T cells.

*Systems Correlations:* Fernández Do Porto et al (40) used Bayesian modelling to compare two competing hypotheses: a direct CD137 signalling model in T cells and an indirect model lacking direct signalling, where increased TNF-α drives T-cell apoptosis. While the indirect model could reproduce much of the data, it failed to capture the effects of CD137 blockade on IFN-γ production and T-cell survival. Thermodynamic integration revealed decisive evidence in favour of the direct signalling model (Bayes factor ≈ 43.7 dB), supporting a central role for CD137’s direct action on T cells.

Taken together, these findings portray CD137 not as a lone actor but as part of an integrated immune network that reflects and potentially shapes how the host responds to *M.tb* infection.

### Role of CD137 as a biomarker for the diagnosis of TB

Two studies assessed CD137 as a potential diagnostic biomarker for TB (**Table 3**). Yan et al (37) used flow cytometry to measure CD137 expression on CD4^+^ T cells in peripheral blood before and after stimulation with the TB-specific antigen CFP-10. They reported a higher percentage of CD137^+^ CD4^+^ T cells in TB patients compared to healthy donors (p<0.0001). The LTBI group also showed higher CD137^+^ CD4^+^ T cells than healthy donors (p=0.0012) but lower than TB patients (p=0.0045). After CFP-10 stimulation, CD137 expression remained unchanged in healthy donors (p>0.05) but increased in TB (p=0.0018) and LTBI (p<0.0001) groups. Another study by Yi et al. (19) measured soluble CD137 (sCD137) levels in blood using ELISA. They found higher sCD137 levels in TB patients compared to healthy controls (p=0.012), with an area under Receiver Operating Characteristics Curve (AUC) of 0.6177 (95% CI: 0.5402–0.6952), sensitivity of 42.45% (95% CI: 34.54–50.76%), and a specificity of 74.68% (95% CI: 64.11–82.97%). Among 139 pulmonary TB (PTB) patients (60 severe [SE], 79 non-severe [non-SE]), sCD137 levels were higher in SE patients (median: 45.01 pg/mL, range: 28.50–70.45) than in non-SE patients (median: 26.82 pg/mL, range: 18.00–42.25; p=0.0056). The ROC curve for distinguishing SE from non-SE patients showed an AUC of 0.7715 (95% CI: 0.6901–0.8529), with a sensitivity of 60.00% (95% CI: 47.37–71.43%) and a specificity of 82.28% (95% CI: 72.42–89.14%), indicating a high potential diagnostic utility.

The elevated levels of CD137 observed in individuals with active TB compared to those latently infected suggest its potential as a biomarker for TB diagnosis and severity when measured as a soluble marker or as a cell surface marker on T cells. Further validation of sCD137 levels may help identify severe TB cases, with CD137 differentiating between TB and LTBI from healthy states.

### Role of CD137 as a prognostic biomarker for TB

Evidence regarding CD137 as a TB prognostic biomarker is scanty. Only one study (19) (**Table 4**) examined its predictive value in relation to treatment outcomes and disease progression. The study assessed sCD137 in 139 plasma samples collected from TB patients based on PTB disease severity and analysed the relationship between the plasma levels of sCD137 and patient prognosis. Of the total study participants, 12 patients died (11 SE, 1 non-SE), and a trend analysis showed sCD137 levels increasing from healthy controls (n=80) to non-SE (n=78), SE (n=49), and deceased patients (n=12; p=0.0128). A log-rank test showed that patients with high sCD137 levels had shorter survival times (p=0.0041).

This finding thus suggests that sCD137 might be associated with poor prognosis with levels increasing with TB disease severity and being highest in patients who died. Given the diagnostic and prognostic potential of CD137, it might equally pose therapeutic potential and worth exploring to harness its full clinical utility.

### Therapeutic role of CD137 in tuberculosis

Two studies explored CD137 as a potential therapeutic target (**Table 5**). A study by Ji et al (39) examined CD137 expression on gamma delta (γδ) T cells in *M.tb* H37Rv-infected PBMCs from TB patients, resisters, and healthy controls. They showed that adoptive transfer of CD137^+^ Vγ2Vδ2 T cells into *M.tb*-infected SCID mice reduced mycobacterial growth in macrophages (p<0.01). Another study by Palma et al (41) found that agonistic anti-4-1BB treatment in Ag85B-immunised mice (NP-mice and p-mice) increased IFN-γ secretion upon CD3ϵ engagement (p<0.01).

Although these findings open a potential space for host-directed therapy, there is still a need for optimisation and intensive safety assessment. Targeting CD137 could thus be a promising immunotherapeutic strategy in TB by enhancing protective immunity and T-cell effector functions.

Taken together, the studies reviewed suggest that CD137 has multifaceted relevance in TB immunology, with early evidence supporting its diagnostic, prognostic, and immunomodulatory potential. However, most findings remain exploratory, and there is a need for multi-centre validation studies in diverse populations to confirm CD137’s clinical utility.

## Discussion

This scoping review synthesises insights from ten studies conducted between 2004 and 2024, shedding light on the potential roles of CD137 in TB. The studies carried out were predominantly from China (n=5), with additional contributions from Europe and South America, outlining current knowledge and identifying gaps across five key themes: molecular mechanisms, interactions with immune markers, and the diagnostic, prognostic, and therapeutic potential of CD137. None of these studies came from Africa, a region of high TB burden and co-morbidities with HIV. This gap culminates in the need for studies from Africa in children and HIV co-infected patients. Notably, studies on the role of CD137 in TB have only recently started gaining momentum, with three of the included studies published in 2024. Across the included studies, we observed consistent signals that CD137 may influence TB pathogenesis and immune regulation. Elevated CD137^+^ CD4^+^ T cells and soluble CD137 were reported in ATB patients, with the potential to distinguish ATB from LTBI and to stratify disease severity. Importantly, higher sCD137 levels correlated with reduced survival, suggesting prognostic value. Functional studies also showed that CD137 expression influences cytokine production, while CD137-targeted interventions reduced mycobacterial growth in murine models. Collectively, these findings position CD137 as both a biomarker and an immunological target with translational promise, though further validation is required to ascertain its translational potential, especially in TB care.

Interestingly, CD137’s expression was confirmed in blood plasma and serum; there is still a need for exploration in more easily accessible samples, such as saliva and urine, that might drive future integration into point-of-care testing.

The molecular mechanisms of CD137 reveal a complex player in TB immunity. This corroborates the findings of Fernández Do Porto et al. and Curto et al, highlighting CD137 induction on monocytes and microglia following mycobacterial stimulation, with changes in staining intensity linked to pathogenicity rather than transcriptional shifts. Also, Martínez Gómez et al (20) and Jiang et al. (38) showed how CD137 influences T cell activation and cytokine production, with changes in TNF-α, IL-6, IL-2, IL-12, and IFN-γ levels depending on infection stage or cell type. Palma et al (41) and Kam et al (34) further demonstrated its role in modulating immune responses, encompassing cytokine secretion to granuloma formation. Overall, despite the urgent need for more clinical translations, these studies highlight the mechanism by which CD137 exerts its response, from its expression to its interaction with its ligand, and finally to the cytokines produced as a result of these interactions, clearly defining CD137’s dual role as both a promoter and regulator of host immune responses.

CD137 doesn’t act alone; rather, it interacts with other immune markers. Kam et al (34) and Ji et al (39) noted correlations with PD-1 and CTLA4, suggesting a connection with immune exhaustion. Palma et al (41) reported cytokine modulation following anti-4-1BB engagement, including suppression of IL-10 and MIP-1β in CD4^+^ T cells, while Fernández Do Porto (40) et al. used Bayesian modelling to dissect competing hypotheses of CD137 function. Their modelling showed that an indirect mechanism, in which TNF-α drives T-cell apoptosis, could replicate much of the data but could not explain the effects of CD137 blockade on IFN-γ production and T-cell survival. Decisive model selection (Bayes factor ≈ 43.7 dB) supported the direct CD137 signalling model, strengthening the case for CD137’s central role in T-cell biology. This positions CD137 within a broader immune environment, potentially amplifying or countering other checkpoint effects, though the full picture requires further investigation. These studies define CD137’s role in both pro- and anti-inflammatory immune tuning, exhibiting synergy or antagonism with PD-1, CTLA4, and IL-10 pathways. This dual behaviour suggests that CD137 may serve as a regulatory node balancing immune activation and tolerance, a role highly relevant in TB pathology, where immune overdrive can worsen outcomes.

Some studies suggest that CD137 may be a promising diagnostic tool for TB. Yan et al. (37) showed that CD137 expression on CD4^+^ T cells, especially after CFP-10 stimulation, differs significantly between active TB, LTBI, and healthy controls, suggesting it could help distinguish these TB disease states. Yi et al (19) added to this by demonstrating elevated soluble CD137 levels in TB patients compared to healthy individuals, with a good diagnostic accuracy and the ability to differentiate severe from non-severe TB. These findings suggest the possibility of utilising CD137 to enhance the stratification of latent TB infection versus active TB infection, especially where traditional methods like IGRA fall short. However, the limited number of studies and lack of large-scale validation across diverse cohorts mean we’re not yet at a point where CD137 can be confidently employed in routine clinical practice. Perhaps, further validation of biosignatures previously identified by Chendi et al (43), or Chegou et al. (44) should incorporate CD137/sCD137 in a larger cohort.

When it comes to prognosis, evidence is scarcer. Study by Yi et al. (19) is the only study that explores this, linking higher sCD137 levels with shorter survival times in pulmonary TB patients, a trend that increases with disease severity. This suggests a possible role for CD137 in predicting outcomes, but the single-study basis and absence of prospective data leave its prognostic utility largely underexplored. More robust, longitudinal studies are needed to determine if CD137 can reliably predict treatment response, risk of relapse, or even death.

Although the therapeutic application of CD137 is still in its early stage, Ji et al (39) demonstrated that transferring CD137^+^ γδ T cells reduced mycobacterial growth in mice, while Palma et al (41) found that anti-4-1BB treatment boosted IFN-γ production in mice. These results are encouraging, but the lack of human trials is still something to consider. Hence, the need for optimisation and intensive safety assessment, and ultimately, integration into clinical trials.

Reflecting on this scoping review, it’s amazing to see CD137’s potential in TB, but the evidence has its limits. With only 10 studies and many with small samples, it’s hard to generalise or conclude the findings. The research is heavily tilted towards Asia, with zero input from high-TB burden regions like Africa, raising questions about its applicability elsewhere. The lack of longitudinal data and human trials also limits our understanding of prognosis and therapy, while unexplored areas like different TB stages or co-infections with diseases such as HIV/AIDs leave gaps. Without assessing a full picture of CD137’s interplay with other markers, and given the field’s early stage, we need larger, standardised, and diverse studies to solidify its clinical utility.

Beyond TB, CD137 has been extensively studied in cancer immunotherapy and autoimmune diseases, where its costimulatory function plays a crucial role in sustaining T cell activation and survival. For example, agonistic anti-CD137 antibodies have been shown to enhance cytotoxic T lymphocyte activity in oncology, while dysregulated CD137 signalling has been implicated in autoimmune pathogenesis (45, 46). These findings underscore the broader immunological significance of CD137 as a modulator of immune responses across various disease contexts. While valuable mechanistic parallels exist, our review deliberately restricts its focus to TB, where the literature remains relatively limited but is rapidly gaining momentum. By situating TB findings within this wider immunological framework, we highlight both the unique and overlapping roles of CD137, without extending beyond the intended scope of this review.

In summary, CD137 emerges as a multifaceted player in TB immunology, spanning diagnostic, prognostic, and therapeutic domains. However, realising its translational potential will require coordinated efforts to validate findings across diverse populations, standardise assay platforms, and explore synergies with existing TB biomarkers. Future studies should concentrate on exploring the diagnostic utility of CD137, emphasising its potential to differentiate between active and latent TB infections and other respiratory diseases, its role as a correlating marker of protection to evaluate vaccine efficacy, and its use in monitoring treatment responses. If successful, CD137 could eventually contribute to an integrated host-based biomarker panel, enhancing personalised approaches to TB diagnosis and treatment.

## Data Availability

The original contributions presented in the study are included in the article/supplementary material. Further inquiries can be directed to the corresponding author.

## References

[1] World Health Organization (WHO) . Global Tuberculosis Report. Geneva: World Health Organization (2024).

[2] Leo S Narasimhan M Rathinam S Banerjee A . Biomarkers in diagnosing and therapeutic monitoring of tuberculosis: a review. Ann Med. (2024) 56:2386030. doi: 10.1080/07853890.2024.2386030, PMID: 39097795 PMC11299445

[3] Norbis L Alagna R Tortoli E Codecasa LR Migliori GB Cirillo DM . Challenges and perspectives in the diagnosis of extrapulmonary tuberculosis. Expert Rev anti-infective Ther. (2014) 12:633–47. doi: 10.1586/14787210.2014.899900, PMID: 24717112

[4] Mandal N Anand PK Gautam S Das S Hussain T . Diagnosis and treatment of paediatric tuberculosis: an insight review. Crit Rev Microbiol. (2017) 43:466–80. doi: 10.1080/1040841X.2016.1262813, PMID: 28502224

[5] Padmapriyadarsini C Narendran G Swaminathan S . Diagnosis & treatment of tuberculosis in HIV co-infected patients. IJMR. (2011) 134:850–65. doi: 10.4103/0971-5916.92630, PMID: 22310818 PMC3284094

[6] Namuganga AR Chegou NN Mayanja-Kizza H . Past and present approaches to diagnosis of active pulmonary tuberculosis. Front Med. (2021) 8:709793. doi: 10.3389/fmed.2021.709793, PMID: 34631731 PMC8495065

[7] Shivakumar P Shettigar KS . Tuberculosis diagnosis: updates and challenges. In: Bacterial Infectious Diseases Annual, vol. 2023. London, United Kingdom: IntechOpen (2022).

[8] Rimal R Shrestha D Pyakurel S Poudel R Shrestha P Rai KR . Diagnostic performance of GeneXpert MTB/RIF in detecting MTB in smear-negative presumptive TB patients. BMC Infect Dis. (2022) 22:321. doi: 10.1186/s12879-022-07287-5, PMID: 35365080 PMC8973748

[9] Piatek AS Van Cleeff M Alexander H Coggin WL Rehr M Van Kampen S . GeneXpert for TB diagnosis: planned and purposeful implementation. Global Health: Sci Practice. (2013) 1:18–23. doi: 10.9745/GHSP-D-12-00004, PMID: 25276513 PMC4168558

[10] Wu J Wang S Lu C Shao L Gao Y Zhou Z . Multiple cytokine responses in discriminating between active tuberculosis and latent tuberculosis infection. Tuberculosis. (2017) 102:68–75. doi: 10.1016/j.tube.2016.06.001, PMID: 28061954

[11] Chegou NN Heyckendorf J Walzl G Lange C Ruhwald M . Beyond the IFN-γ horizon: biomarkers for immunodiagnosis of infection with Mycobacterium tuberculosis. Eur Respir J. (2014) 43:1472–86. doi: 10.1183/09031936.00151413, PMID: 24311770

[12] Kahwati LC Feltner C Halpern M Woodell CL Boland E Amick HR . Primary care screening and treatment for latent tuberculosis infection in adults: evidence report and systematic review for the US Preventive Services Task Force. Jama. (2016) 316:970–83. doi: 10.1001/jama.2016.10357, PMID: 27599332

[13] Carranza C Pedraza-Sanchez S de Oyarzabal-Mendez E Torres M . Diagnosis for latent tuberculosis infection: new alternatives. Front Immunol. (2020) 11:2006. doi: 10.3389/fimmu.2020.02006, PMID: 33013856 PMC7511583

[14] Mayer-Barber KD Barber DL . Innate and adaptive cellular immune responses to Mycobacterium tuberculosis infection. Cold Spring Harb Perspect Med. (2015) 5:a018424. doi: 10.1101/cshperspect.a018424, PMID: 26187873 PMC4665043

[15] González-Juarrero M O’Sullivan MP . Optimization of inhaled therapies for tuberculosis: the role of macrophages and dendritic cells. Tuberculosis. (2011) 91:86–92. doi: 10.1016/j.tube.2010.08.007, PMID: 20888298

[16] Jasenosky LD Scriba TJ Hanekom WA Goldfeld AE . T cells and adaptive immunity to Mycobacterium tuberculosis in humans. Immunol Rev. (2015) 264:74–87. doi: 10.1111/imr.12274, PMID: 25703553

[17] Simmons JD Stein CM Seshadri C Campo M Alter G Fortune S . Immunological mechanisms of human resistance to persistent Mycobacterium tuberculosis infection. Nat Rev Immunol. (2018) 18:575–89. doi: 10.1038/s41577-018-0025-3, PMID: 29895826 PMC6278832

[18] Hashimoto K . CD137 as an attractive T cell co-stimulatory target in the TNFRSF for immuno-oncology drug development. Cancers. (2021) 13:2288. doi: 10.3390/cancers13102288, PMID: 34064598 PMC8150789

[19] Yi L Yan J Wei P Long S Wang X Gu M . The levels of soluble CD137 are increased in tuberculosis patients and associated with disease severity and prognosis. Eur J Immunol. (2024) 54:e2350796. doi: 10.1002/eji.202350796, PMID: 38922884

[20] Martínez Gómez JM Koh VH Yan B Lin W Ang ML Rahim SZ . Role of the CD137 ligand (CD137L) signaling pathway during Mycobacterium tuberculosis infection. Immunobiol. (2014) 219:78–86. doi: 10.1016/j.imbio.2013.08.009, PMID: 24091276

[21] Gremo F Presta M . Role of fibroblast growth factor-2 in human brain: a focus on development. Int J Dev Neurosci. (2000) 18:271–9. doi: 10.1016/S0736-5748(99)00095-7, PMID: 10715581

[22] Hurtado JC Kim SH Pollok KE Lee ZH Kwon BS . Potential role of 4-1BB in T cell activation. Comparison with the costimulatory molecule CD28. J Immunol. (1995) 155:3360–7. doi: 10.4049/jimmunol.155.7.3360, PMID: 7561030

[23] Rogers PR Dubey C Swain SL . Qualitative changes accompany memory T cell generation: faster, more effective responses at lower doses of antigen. J Immunol. (2000) 164:2338–46. doi: 10.4049/jimmunol.164.5.2338, PMID: 10679068

[24] Lee HW Park SJ Choi BK Kim HH Nam KO Kwon BS . 4-1BB promotes the survival of CD8+ T lymphocytes by increasing expression of Bcl-xL and Bfl-1. J Immunol. (2002) 169:4882–8. doi: 10.4049/jimmunol.169.9.4882, PMID: 12391199

[25] Sun Y Chen JH Fu Y . Immunotherapy with agonistic anti-CD137: two sides of a coin. Cell Mol Immunol. (2004) 1:31–6., PMID: 16212918

[26] Wang C Lin GH McPherson AJ Watts TH . Immune regulation by 4-1BB and 4-1BBL: complexities and challenges. Immunol Rev. (2009) 229:192–215. doi: 10.1111/j.1600-065X.2009.00765.x, PMID: 19426223

[27] Zhu Y Chen L . CD137 as a biomarker for tumor-reactive T cells: finding gold in the desert. Clin Cancer Res. (2014) 20:3–5. doi: 10.1158/1078-0432.CCR-13-2573, PMID: 24166912

[28] Menk AV Scharping NE Rivadeneira DB Calderon MJ Watson MJ Dunstane D . 4-1BB costimulation induces T cell mitochondrial function and biogenesis enabling cancer immunotherapeutic responses. J Exp Med. (2018) 215:1091–100. doi: 10.1084/jem.20171068, PMID: 29511066 PMC5881463

[29] Teijeira A Labiano S Garasa S Etxeberria I Santamaría E Rouzaut A . Mitochondrial morphological and functional reprogramming following CD137 (4-1BB) costimulation. Cancer Immunol Res. (2018) 6:798–811. doi: 10.1158/2326-6066.CIR-17-0767, PMID: 29678874

[30] Fernández Do Porto DA Jurado JO Pasquinelli V Alvarez IB Aspera RH Musella RM . CD137 differentially regulates innate and adaptive immunity against Mycobacterium tuberculosis. Immunol Cell Biol. (2012) 90:449–56. doi: 10.1038/icb.2011.63, PMID: 21747409 PMC3330265

[31] Curto M Reali C Palmieri G Scintu F Schivo ML Sogos V . Inhibition of cytokines expression in human microglia infected by virulent and non-virulent mycobacteria. Neurochem Int. (2004) 44:381–92. doi: 10.1016/j.neuint.2003.08.012, PMID: 14687603

[32] Thum E Shao Z Schwarz H . CD137, implications in immunity and potential for therapy. Front Biosci. (2009) 14:4173–88. doi: 10.2741/3521, PMID: 19273343

[33] Yi L Jin X Wang J Yan Z Cheng X Wen T . CD137 agonists targeting CD137-mediated negative regulation show enhanced antitumor efficacy in lung cancer. Front Immunol. (2022) 13:771809. doi: 10.3389/fimmu.2022.771809, PMID: 35197968 PMC8859117

[34] Kam NW Lo AWI Hung DTY Ko H Wu KC Kwong DLW . Shift in tissue-specific immune niches and CD137 expression in tuberculoma of pembrolizumab-treated nasopharyngeal carcinoma patients. Cancers. (2024) 16:268. doi: 10.3390/cancers16020268, PMID: 38254759 PMC10813936

[35] Lee KY Mei Y Liu H Schwarz H . CD137-expressing regulatory T cells in cancer and autoimmune diseases. Mol Ther. (2025) 33:51–70. doi: 10.1016/j.ymthe.2024.12.010, PMID: 39668561 PMC11764688

[36] Glorieux C Huang P . CD137 expression in cancer cells: regulation and significance. Cancer Commun. (2019) 39:1–3. doi: 10.1186/s40880-019-0386-4, PMID: 31703738 PMC6842176

[37] Yan ZH Zheng XF Yi L Wang J Wang XJ Wei PJ . CD137 is a useful marker for identifying CD4(+) T cell responses to mycobacterium tuberculosis. Scand J Immunol. (2017) 85:372–80. doi: 10.1111/sji.12541, PMID: 28218958

[38] Jiang J Cao Z Shan W Liu H Cheng X . 4-1BB expression on MAIT cells is associated with enhanced IFN-γ production and depends on IL-2. Cell Immunol. (2018) 328:58–69. doi: 10.1016/j.cellimm.2018.03.013, PMID: 29631725

[39] Ji X Huang G Peng Y Wang J Cai X Yang E . CD137 expression and signal function drive pleiotropic γδ T-cell effector functions that inhibit intracellular M. tuberculosis growth. Clin Immunol. (2024) 266:110331. doi: 10.1016/j.clim.2024.110331, PMID: 39067675

[40] Fernández Do Porto DA Auzmendi J Peña D García VE Moffatt L . Bayesian approach to model CD137 signaling in human M. tuberculosis *in vitro* responses. PloS One. (2013) 8:e55987. doi: 10.1371/journal.pone.0055987, PMID: 23437083 PMC3577821

[41] Palma C Vendetti S Cassone A . Role of 4-1BB receptor in the control played by CD8(+) T cells on IFN-gamma production by Mycobacterium tuberculosis antigen-specific CD4(+) T Cells. PloS One. (2010) 5:e11019. doi: 10.1371/journal.pone.0011019, PMID: 20544034 PMC2882340

[42] Mo W Cui Z Zhao J Xian X Huang M Liu J . The predictive value of TNF family for pulmonary tuberculosis: a pooled causal effect analysis of multiple datasets. Front Immunol. (2024) 15:1398403. doi: 10.3389/fimmu.2024.1398403, PMID: 38835752 PMC11148272

[43] Chendi BH Snyders CI Tonby K Jenum S Kidd M Walzl G . A plasma 5-marker host biosignature identifies tuberculosis in high and low endemic countries. Front Immunol. (2021) 12. doi: 10.3389/fimmu.2021.608846, PMID: 33732236 PMC7958880

[44] Chegou NN Sutherland JS Malherbe S Crampin AC Corstjens PL Geluk A . Diagnostic performance of a seven-marker serum protein biosignature for the diagnosis of active TB disease in African primary healthcare clinic attendees with signs and symptoms suggestive of TB. Thorax. (2016) 71:785–94. doi: 10.1136/thoraxjnl-2015-207999, PMID: 27146200

[45] Vinay DS Kwon BS . Immunotherapy of cancer with 4-1BB. Mol Cancer Ther. (2012) 11:1062–70. doi: 10.1158/1535-7163.MCT-11-0677, PMID: 22532596

[46] Chester C Sanmamed MF Wang J Melero I . Immunotherapy targeting 4-1BB: mechanistic rationale, clinical results, and future strategies. Blood. (2018) 131:49–57. doi: 10.1182/blood-2017-06-741041, PMID: 29118009

